# Effects of Hydroxychloroquine on endOthelial function in eLDerly with sleep apnea (HOLD): study protocol for a randomized clinical trial

**DOI:** 10.1186/s13063-021-05610-0

**Published:** 2021-09-17

**Authors:** Leticia Maria Tedesco Silva, Antonio Cortes, Beatriz Rossi, Liliana Boll, Gustavo Waclawovsky, Bruna Eibel, Sandro Cadaval Gonçalves, Maria Claudia Irigoyen, Denis Martinez

**Affiliations:** 1 Universidade Federal do Rio Grande do Sul, Porto Alegre, Brazil; 2 Instituto de Cardiologia - Fundação Universitária de Cardiologia (IC-FUC), Porto Alegre, Brazil; 3 Universidade de São Paulo, São Paulo, Brazil

**Keywords:** Hydroxychloroquine, Endothelial function, Sleep apnea

## Abstract

**Background:**

Sleep apnea and coronary artery disease are prevalent and relevant diseases. The mechanism by which sleep apnea leads to coronary artery disease remains unclear. Intermittent hypoxia, caused by sleep apnea, leads to inflammation and consequent endothelial dysfunction. Endothelial dysfunction precedes the development of atherosclerotic disease and the occurrence of cardiovascular events. Agents that potentially act to improve endothelial function can help prevent cardiovascular events. Patients using immunomodulators due to rheumatic diseases have a lower prevalence of cardiovascular diseases. However, the potential cardioprotective effect of these drugs in patients without autoimmune diseases is not clear. Hydroxychloroquine (HCQ) is an immunomodulator used to treat rheumatoid arthritis and systemic lupus erythematosus. In addition to its anti-inflammatory properties, HCQ reduces cholesterol and blood glucose levels and has antithrombotic effects. The drug is inexpensive and widely available. Adverse effects of HCQ are rare and occur more frequently with high doses.

**Objective:**

In this randomized clinical trial, the effect of HCQ treatment on endothelial function will be tested in seniors with sleep apnea.

**Methods:**

We will recruit participants over the age of 65 and with moderate-severe sleep apnea from an ongoing cohort. We chose to use this sample already evaluated for sleep apnea for reasons of convenience, but also because the elderly with sleep apnea are vulnerable to heart disease. Endothelial function will be assessed by examining flow-mediated dilation of the brachial artery, the gold standard method, considered an independent predictor of cardiovascular events in the general population and by peripheral arterial tonometry, the most recent and most easily obtained method. Hydroxychloroquine will be used at a dose of 400 mg/daily for 8 weeks.

**Discussion:**

Our study aims to obtain evidence, albeit preliminary, of the efficacy of hydroxychloroquine in improving endothelial function and reducing cardiovascular risk markers. If the improvement occurs, we plan to design a randomized multicenter clinical trial to confirm the findings.

**Trial registration:**

ClinicalTrials.gov NCT04161339. Registered on November 2019.

## Administrative information

The order of the items has been modified to group similar items (see http://www.equator-network.org/reporting-guidelines/spirit-2013-statement-defining-standard-protocol-items-for-clinical-trials/).

**Table:** 

Title {1}	Effects of Hydroxychloroquine on endOthelial function in eLDerly with sleep apnea (HOLD): study protocol for a randomized clinical trial
Trial registration {2a and 2b}.	ClinicalTrials.gov, ID: NCT04161339. Registered on November, 2019. Anti-Inflammatory Drug and Endothelial Function (HOLD)
Protocol version {3}	March 2021, version 1.0
Funding {4}	This study was financed in part by the Coordenação de Aperfeiçoamento de Pessoal de Nível Superior -Brasil (CAPES) - Finance Code 001 and by the Fundação de Amparo à Pesquisa do Rio Grande do Sul (FAPERGS)
Author details {5a}	Leticia Maria Tedesco Silva^1^, Antonio Cortes^1^, Beatriz Rossi^2^, Liliana Boll^2^, Gustavo Waclawosky^2^, Bruna Eibel^2^, Sandro Gonçalves^1^, Maria Claudia Irigoyen^2-3^, Denis Martinez^1^ ^1^ - Universidade Federal do Rio Grande do Sul ^2^ - Instituto de Cardiologia - Fundação Universitária de Cardiologia (IC-FUC) ^3^ - Universidade de São Paulo
Name and contact information for the trial sponsor {5b}	FAPERGS - Fundação de Amparo à Pesquisa do Estado do Rio Grande do Sul Address: Av. Borges de Medeiros, 261 - 2° andar - Centro Histórico, Porto Alegre - RS, 90020-021, Brazil Phone: +55 51 3221-4922
Role of sponsor {5c}	The study sponsor had no role in the design, collection, management, analysis or interpretation of data, nor in the writing of the report.
Committees {5d}	**Principal Investigator and research physician **Design and conduct of HOLD Preparation of protocol and revisions Publication of study reports **Data management** Biostatistician affiliated to Hospital de Clinicas de Porto Alegre **Ethical committee of Hospital de Clinicas de Porto Alegre** Composed of individuals with scientific and medical expertise Agreement of final protocol Review progress of study and if necessary approving alterations to the protocol Ensure the rights and welfare of human subjects are protected Ensure the study is conducted in compliance with the approved study protocol and federal regulations Ensure data validity and integrity. Due to its small size and restricted funding, the study did not have a coordinating centre, steering committee, endpoint committee or data management team.

## Introduction

### Background and rationale {6a}

#### Obstructive sleep apnea (OSA) and cardiovascular disease

In a meta-analysis, the prevalence of sleep apnea in the general adult population ranges from 6% to 17%, reaching 49% at advanced ages [1]. In a Brazilian epidemiological study, OSA affected 80 to 95% of the elderly [2]. The most damaging consequence of OSA is an increase in cardiovascular morbidity and mortality [3]. Cardiovascular diseases are responsible for 20% of deaths of Brazilians over 30 years old and lead to increased morbidity and mortality in over 30% of the elderly [4]. Prospective population studies have shown that individuals with severe untreated sleep apnea have a higher risk of general and cardiovascular mortality, independent of traditional cardiovascular risk factors [5]. Apnea patients, compared to the general population, are twice as likely to have hypertension [6], ischemic heart disease [7], and cerebrovascular disease [8]. In 2004, the National Heart, Lung, and Blood Institute committee proposed lines of research to enlighten the consequences of sleep disorders in heart disease [9]. In 2008, the American Heart Association and the American College of Cardiology published a consensus indicating the need for researches like ours that addresses the major cardiovascular consequences of sleep apnea [10]. The main reasons why sleep apnea is associated with cardiovascular diseases are the periods of intermittent hypoxia and the repeated nocturnal awakenings precipitated by apneas. Apnea occurs when anatomical and/or functional changes in the airway, associated with loss of tone in the pharyngeal dilator muscles during sleep, lead to airway collapse and interruption of airflow. Nighttime awakenings cause chronic sympathetic hyperactivity and intermittent hypoxia causes oxidative stress, which causes inflammation, the onset of endothelial dysfunction and, later, atherosclerosis. The association between coronary artery disease and sleep apnea appears to be consistent. In a cross-sectional analysis of the Sleep Heart Health Study cohort, individuals in the highest quartile of apnea-hypopnea index present a 27% risk increase for coronary artery disease. Sleep apnea increases the chance of having ischemic heart disease by 65% [11] and having a heart attack by 71% [12]. Our group demonstrated that sleep apnea is a more robust risk factor for coronary artery disease than classic factors such as cholesterol, in a sample excluding morbidly obese, diabetic, and smokers [13].

#### Endothelial function

The endothelium, by isolated area, is one of the largest organs in the body, composed of trillions of cells, weighing more than 1 kg and covering almost 3 m^2^ in a 70-kg adult male [14]. It interacts with multiple systems and has been implicated in the pathogenesis of neurological, renal, liver, vascular, dermatological, immunological, and cardiovascular diseases. It is a highly specialized tissue, responsible for vascular homeostasis through the regulation of arteriolar tone, platelet aggregation, smooth muscle cell growth, and leukocyte adhesion. The endothelium regulates the vascular tone through the secretion vasodilator, such as nitric oxide (NO), and vasoconstrictors, such as endothelin. Damage to the endothelium and dysfunction of this tissue are associated with the development of arterial hypertension and atherosclerosis. There are many molecular and cellular mechanisms involved with endothelial damage and vascular aging. Since inflammation and oxidative stress are part of these mechanisms, tackling these two processes can be considered as future therapeutic targets for the prevention of cardiovascular disease [15]. A review article on the topic states that a greater understanding of endothelial function provides not only a grasp of the pathophysiology of cardiovascular disease but also an opportunity for clinical treatment, early detection of diseases, stratification of cardiovascular risk, and evaluation of therapeutic response [16].

#### Flow-mediated dilatation of the brachial artery

The assessment of flow-mediated dilation (FMD) was first introduced in the 1990s as a non-invasive approach to examining vasodilator function in vivo. The FMD result quantifies the endothelium-dependent arterial function mediated by nitric oxide, being used as an indirect marker of vascular health [17]. A meta-analysis that included 32 studies and 15,000 individuals concluded that the FMD result is an independent predictor of cardiovascular events and death. Each 1% dilation increase in the FMD result was associated with a 10% lower risk of cardiovascular event or death. In that study, the predictive effect of brachial FMD was more substantial for cardiovascular mortality than for general mortality, suggesting that impaired endothelial function is predominantly a cardiovascular risk factor [18].

The method has limitations that must be considered. Small changes in the methodological approach can critically influence results and decrease exam reproducibility. Therefore, the compliance of guidelines with updated and standardized methodology is indicated to reduce measurement errors and improve FMD reliability in clinical studies [19].

#### Peripheral arterial tonometry

Peripheral arterial tonometry (PAT) is a non-invasive method of assessing endothelial function in clinical practice. The method was incorporated into several population and multicenter studies, such as the Framingham Heart Study. The results are based on digital pulse amplitude variation during reactive hyperemia induced by a 5-min forearm cuff occlusion. It is operator/interpreter independent and provides immediate results, which are advantages compared to FMD. The reactive hyperemia index (RHI) obtained by PAT is considered to correlate significantly with coronary endothelial function [20]. PAT is a predictor of cardiovascular risk in high-risk patients, according to several authors [21–24]. The method also has good reproducibility, according to studies by Brant et al and Reisner et al. [25] In two large population studies, totaling more than 10,000 participants, there is only a modest correlation between FMD and PAT [26, 27]. Therefore, both methods will be used in this study, in order to quantify aspects of both macro- and microvascular circulation and compare the two methods.

#### Endothelial function and obstructive sleep apnea

Sleep apnea is related to systemic inflammation, oxidative stress, and endothelial dysfunction [28, 29]. These factors are protagonists in the process that leads patients with sleep apnea to develop cardiovascular disease. The association between endothelial dysfunction and sleep apnea was initially considered ambiguous because there are numerous common features among patients with endothelial dysfunction and sleep apnea. Age, obesity, smoking, and alcohol consumption were factors cited as potential causes of the two conditions, confusing a possible cause-effect relationship. A meta-analysis by Wang et al. concluded that moderate/severe sleep apnea was significantly associated with endothelial dysfunction, increased arterial stiffness and increased serum levels of inflammatory markers. The meta-regression data suggest that the adverse effect of moderate-severe sleep apnea on endothelial function is not modified by potential confounders, such as body mass index [30]. Cross-sectional analyses of population studies and case-control studies consistently demonstrated an association between obstructive sleep apnea and impaired endothelium-dependent vasodilation [31]. In vitro experiments demonstrate that the serum of patients with sleep apnea impairs the migration of coronary endothelial cells [32]. Our group published studies demonstrating that intermittent hypoxia causes oxidative stress [33], inflammation, and damage to lipids and proteins [34].

#### Hydroxychloroquine

Hydroxychloroquine was initially an antimalarial drug. Due to its anti-inflammatory properties, it is commonly used to treat rheumatic diseases, in particular rheumatoid arthritis and connective tissue diseases such as systemic lupus erythematous. HCQ reduces the activation of the innate immunity system by inhibiting the stimulation of Toll-like receptors [35], which can play an important role in the activation of inflammatory cells in atherosclerotic patients [36]. Some studies suggest that hydroxychloroquine also reduces the production of cytokines important in the pathogenesis of atherosclerosis, such as interleukin-1 and 6 and tumor necrosis factor alpha (TNF-α) [37, 38]. TNF-α block therapy was associated to a risk reduction of coronary artery disease among patients with rheumatoid arthritis [39].

In addition to its anti-inflammatory effects, HCQ has other properties that may be beneficial in the treatment of coronary artery disease [40]. In patients with lupus, chloroquine inhibits the synthesis of several members of the matrix metalloproteinase family, especially MMP-9 [41], enzymes capable of degrading interstitial collagen in the fibrotic layer of the atherosclerotic plaque [42].

Cohort studies have shown that HCQ lowers cholesterol in patients with lupus and rheumatoid arthritis [43–46]. In addition, in rheumatoid arthritis, the use of HCQ has been associated with a lower incidence of type 2 diabetes [47–49], lower levels of glycosylated hemoglobin (HbA1c) [50], and better sensitivity to insulin and beta cell function [51]. In two randomized studies of diabetics with inadequate glycemic control, HCQ significantly reduced glucose levels [52, 53].

In addition, HCQ may have antithrombotic properties [54, 55]. In mice, HCQ reduced the size and duration of the thrombus and caused a decrease in the thickness of the vascular wall and the progression of atherosclerosis [56, 57]. In a case-control study, among patients with lupus, the use of HCQ was associated with a reduced risk of thromboembolic complications [58].

Sharma et al. reported in a retrospective study involving 1266 patients with rheumatoid arthritis an association between HCQ use and a 72% reduced risk of an outcome composed of acute coronary syndrome, cardiac revascularization, stroke, transient ischemic accident, peripheral arterial disease, and sudden death [59].

Evidence from studies in patients with rheumatic diseases converges to a role for HCQ in reducing cardiovascular risk. However, its cardiovascular effects in patients at higher cardiovascular risk but without rheumatic diseases are unknown.

Some common side effects of hydroxychloroquine, occurring in 1-10% of users, include anorexia, emotional lability, headache, blurred vision, abdominal pain, nausea, rash, and itching. Rarely, in 0.1–1% of high-dose users, Stevens-Johnson syndrome, cardiomyopathy, and retinopathy may occur.

#### Overall

Considering that sleep apnea is a chronic disease with an inflammatory component, as well as rheumatic conditions, HCQ, due to its favorable effects in the reduction of several cardiovascular risk factors, may improve endothelial dysfunction associated with sleep apnea.

Considering the low cost of HCQ, the high prevalence of sleep apnea in the elderly, and the high morbidity and mortality of cardiovascular diseases, the search for feasible approaches in primary care that can reduce these outcomes is justified. Thus, the use of HCQ in patients with sleep apnea and at high risk for coronary artery disease represents an entirely new approach to this disease.

### Objectives {7}

#### Main objective

To test in a randomized clinical trial the effect of hydroxychloroquine on endothelial function and its correlates, in elderly people with sleep apnea.

#### Specific objectives

- To test in a randomized clinical trial the effect of hydroxychloroquine on endothelial function measured by peripheral arterial tonometry.
- To test in a randomized clinical trial the effect of hydroxychloroquine on endothelial function measured by flow-mediated dilation of the brachial artery.
- Compare the endothelial function measured by peripheral arterial tonometry with the endothelial function measured by the mediated flow dilation of the brachial artery in the same individuals to support assessments of the quality of the methods
- Test in a randomized clinical trial the effect of hydroxychloroquine on inflammatory markers such as reactive C-protein.
- Test in a randomized clinical trial the effect of hydroxychloroquine on glycemic homeostasis, assessed by fasting glucose and HbA1c levels.
- Test in a randomized clinical trial the effect of hydroxychloroquine on the lipid profile
- To test in a randomized clinical trial the effect of hydroxychloroquine on the apnea-hypopnea index and the mean and minimum O_2_ saturations assessed by a portable sleep monitoring device

### Trial design {8}

This exploratory study was designed as a randomized researcher and patient blinded controlled trial with a primary endpoint of change in endothelial function after 8 weeks of treatment with hydroxychloroquine. Randomization will be performed as block randomization with a 1:1 allocation ratio.

## Methods: Participants, interventions, and outcomes

### Study setting {9}

The study is conducted in the research laboratory of two academic hospitals in Porto Alegre, Brazil: Hospital de Clínicas de Porto Alegre and Instituto de Cardiologia do Rio Grande do Sul.

### Eligibility criteria {10}

#### Inclusion criteria

- People over 65 years old
- Apnea-hypopnea index greater than 15 events per hour

#### Exclusion criteria

- Contraindication to the use of hydroxychloroquine (porphyria, retinopathy, severe hepatic or renal dysfunction, neuropathy and/or muscle disease).
- Rheumatic diseases
- Chronic infections
- Serious, terminal, or disabling disease
- Previous ECG with evidence of long QT interval
- Previous or current treatment for obstructive sleep apnea

### Who will take informed consent? {26a}

Members of our research group, formed by nurses, physical educators, nutritionists, medical doctors, and physiotherapists, will obtain informed consent from potential trial participants. After a brief explanation of the study made by phone, we will schedule an appointment for those who are willing to participate. In this appointment, all aspects and details of the study will be explained to the potential participant and the informed written consent will be signed and given a copy to the participant.

## Interventions

### Explanation for the choice of comparators {6b}

Since there is no standard treatment available for endothelial dysfunction, we decided to compare hydroxychloroquine with placebo. The placebo group will receive capsules identical to those of hydroxychloroquine, containing the same excipient as the industrialized product.

### Intervention description {11a}

The intervention group will receive a 400 mg hydroxychloroquine capsule daily for 8 weeks, to be taken with food or milk during lunchtime. Hydroxychloroquine is generally prescribed at a daily dose of 6.5 mg (or less) per kg of body weight (using this formula, the dosage for a 70-kg person would be 455 mg/day). Considering patients newly diagnosed with lupus take 400 mg once daily, we decided to use the same dose due to its known safety and efficacy.

### Criteria for discontinuing or modifying allocated interventions {11b}

The intervention will be discontinued in case of any severe adverse effect or withdrawal of participant consent

### Strategies to improve adherence to interventions {11c}

Every 2 weeks after starting the intervention, participants will receive a call to monitor adherence and adverse effects. At the 8-week follow-up visit, participants will have their unused capsules counted and recorded on the case report form.

### Relevant concomitant care permitted or prohibited during the trial {11d}

The participants are advised to not initiate any new care during the trial. The participant will be excluded from the trial if any new concomitant care needs to be initiated.

### Outcomes {12}

#### Outcomes

##### Main outcome

- Difference between the two groups on endothelial function measured by FMD (change of percentage of dilatation of brachial artery diameter from baseline) and PAT (change of reactive hyperemia index of finger from baseline) after 8 week of intervention.

##### Secondary outcomes

- Difference between the two groups on changes from baseline in the blood levels of C-reactive protein
- Difference between the two groups on changes from baseline in the blood levels of HbA1c fraction
- Difference between the two groups on changes from baseline in blood levels of fasting glucose
- Difference between the two groups on changes from baseline in lipid profile (blood levels of fasting total cholesterol, high-density lipoprotein cholesterol, and triglycerides)
- Difference between the two groups on changes from baseline in the apnea-hypopnea index and in the mean and minimum oxygen saturation measured with the portable sleep monitoring device
- Difference between the two groups on changes from baseline in weight
- Difference between the two groups on changes from baseline in systolic blood pressure and diastolic blood pressure
- Comparison between the rate of adverse effects on intervention × control group.

### Participant timeline {13}

**Fig. 1:**
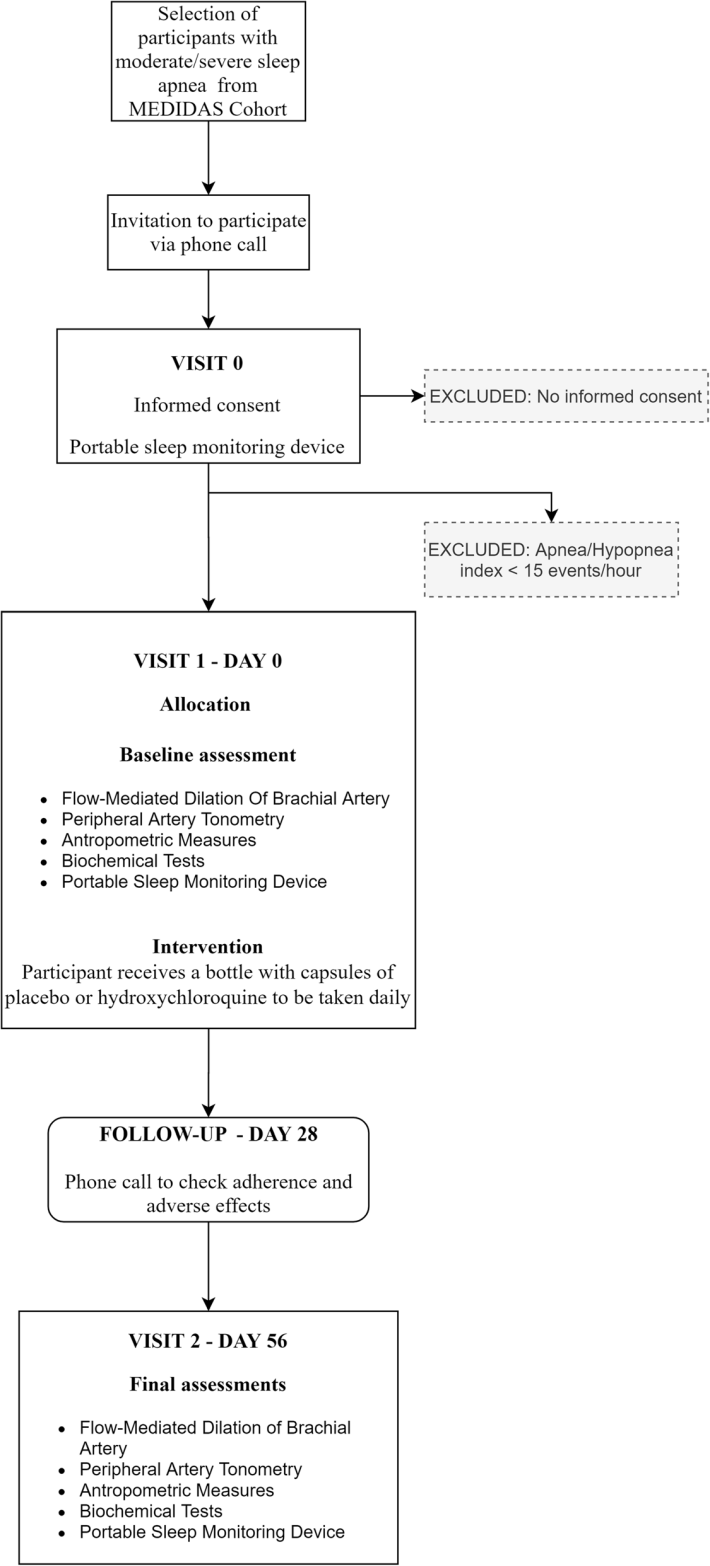
Time schedule of the study

Figure 1 shows the time schedule of the study.

### Sample size {14}

Based on the effect size estimate of 0.3 standard deviations in the response of endothelial function after HCQ, for a 95% power and 0.05 level of alpha error probability, the sample size was estimated at 20 participants per group. Allowing for extra recruitment to compensate for losses, the sample size will be 25 participants per group, with a sum of 50 individuals.

### Recruitment {15}

Patients will be recruited by telephone. We will contact participants in the MEDIDAS cohort study who meet the inclusion criteria for this study. The MEDIDAS cohort study is composed by elderly with different degrees of obstructive sleep apnea, recruited from a local basic health unit, after positive screening for sleep apnea symptoms.

## Assignment of interventions: allocation

### Sequence generation {16a}

Participants will be randomly assigned to either control or experimental group with a 1:1 allocation as per a computer-generated allocation list (https://www.randomizer.org/) using permuted blocks of four.

### Concealment mechanism {16b}

The bottles with hydroxychloroquine or placebo will be numbered in the handling pharmacy that will produce the capsules. Only the pharmacist will have access to the allocation list. Each participant will match the bottle with the number that the participant will receive in the plan. Both patients and researchers involved with either primary or secondary outcome measures will be blinded to the patient’s allocation.

### Implementation {16c}

Someone from outside the research team will generate the allocation sequence.

## Assignment of interventions: Blinding

### Who will be blinded {17a}

Trial participants, researchers involved in the study, outcome assessors will be blinded.

### Procedure for unblinding if needed {17b}

Unblinding will be permissible in case of severe adverse effects. The pharmacist with the allocation list will be contacted to inform the assigned group of the participant in question.

## Data collection and management

### Plans for assessment and collection of outcomes {18a}

#### Biochemical tests

Biochemical assessment will be performed on all participants at the beginning of the study and after 8 weeks of treatment. The exams will be held early in the morning, with participants fasting. Blood samples will be collected in five different sampling tubes with a capacity of 9 ml. The samples will be taken to the laboratory to measure plasma levels of ultra-sensitive C-reactive protein, total cholesterol, HDL-cholesterol, triglycerides, glucose, and glycated hemoglobin.

#### Peripheral arterial tonometry

The microvascular endothelial function will be determined by an automatic device (EndoPAT2000, Itamar Medical, Cesarea, Israel), with the technique already described. The cuff will be placed on the non-dominant arm, 2 cm above the cubital fossa, and RH-PAT tests on the pulp of each index finger will be performed. The basal pulse amplitude will be measured for 5 min. The arterial blood flow will be interrupted on one side for 5 min by insufflation of the cuff, with an occlusion pressure of 200 mmHg, or 60 mmHg above systolic blood pressure. After 5 min of occlusion, the cuff is deflated and reactive hyperemia, the RH-PAT sign, is registered in both hands for an additional 5 min. The contralateral finger is a control for changes in systemic vasodilation. The reactive hyperemia index (RHI) is automatically calculated by the RH-PAT equipment according to the manufacturer’s formula. RHI is defined as the ratio between post-deflation pulse amplitude, 90 to 150 s after the cuff is released, and the mean baseline pulse amplitude. This result is divided by the corresponding proportion of the control finger, and multiplied by a correction factor in relation to the baseline. Low RHI values are related to an inadequate endothelial vasodilator response.

#### Flow-mediated dilation of the brachial artery

High-resolution ultrasound equipment (Esaote) will be used to assess arterial endothelial function. A high frequency transducer will be used to obtain longitudinal images of the brachial artery walls. The images of diameter and arterial flow, at each moment of the protocol, will be recorded simultaneously on a computer with the aid of a capture card (Easycap). To minimize operational errors, both the transducer and the subject's arm will be positioned and maintained in the same position during the examination. Baseline images will be recorded for 1 min and then the pressure cuff on the forearm will be inflated to 200 mmHg and maintained for 5 min, characterizing reactive hyperemia. After deflating the cuff, 3 min of endothelium-dependent brachial artery dilation will be recorded. After the exam, the images recorded in MPEG will be converted to MP4 and then analyzed using specific software (Cardiovascular Suite) for the diameter and arterial flow before and after reactive hyperemia.

#### Portable sleep monitoring device

The portable sleep monitoring device Somnocheck Micro (Weinmann Medical Technology, Hamburg, Germany) will be attached to the patient’s wrist. The patient will also use a nasal cannula to register airflow. The device will be programmed to turn on at 22 pm and turn off at 10 am. A combination of photoplethysmography-derived pulse wave analysis and respiratory flow signals may enable differentiation between obstructive and central apnea and provide information regarding the extent of sleep fragmentation. In detail, respiratory effort derives by analyzing fluctuations of the pulse wave analysis signal caused by intrathoracic pressure changes during spontaneous breathing cycles. Central apneas, RERAs, mean and minimum oxygen saturation, and the apnea-hypopnea index will be reported according to the rules of the American Academy of Sleep Medicine. The portable sleep monitoring exam will be performed before and after 8 weeks of treatment.

### Plans to promote participant retention and complete follow-up {18b}

In order to promote participant retention and complete follow-up, all participants will be asked to provide, at the beginning of the study, their updated contact information.

### Data management {19}

The web-based application REDCap will be used for data entry and storage. Original study forms will be entered and kept on file at the participating site. Participant files will be maintained in storage for 2 years after completion of the study.

### Confidentiality {27}

All study-related information will be stored securely at the study site. All participant information will be stored in locked file cabinets in areas with limited access. All laboratory specimens, reports, data collection, process, and administrative forms will be identified by a coded ID [identification] number only to maintain participant confidentiality. All records that contain names or other personal identifiers, such as locator forms and informed consent forms, will be stored separately from study records identified by code number.

## Statistical methods

### Statistical methods for primary and secondary outcomes {20a}

Normally distributed data will be presented as mean and standard deviation. Data not normally distributed will be presented as median and interquartile range. The natural logarithmic transformation of the variable will be used to correct its non-normal distribution in analyses that assume the normal data distribution. The intervention arm (hydroxychloroquine) will be compared against the control for all primary analysis. We will use chi-squared test for binary outcomes, and *T* test for continuous outcomes. We will examine the residual to assess model assumptions and goodness-of-fit. The group × time interaction will be tested using generalized estimating equations. We will use the Bonferroni method to appropriately adjust the overall level of significance for multiple primary outcomes, and secondary outcomes. Results with a probability < 0.05 of alpha error will be considered statistically significant. Statistical analyses will be performed using SPSS software (SPSS Inc., Chicago, IL, USA).

### Interim analyses {21b}

No interim analysis will be performed.

### Methods for additional analyses (e.g., subgroup analyses) {20b}

There is no plan to conduct any subgroup or adjusted analyses.

### Methods in analysis to handle protocol non-adherence and any statistical methods to handle missing data {20c}

Participants who withdraw consent for continued follow-up will be included in the analysis by modern imputation methods for missing data. The effect that any missing data might have on results will be assessed via sensitivity analysis of augmented data sets.

## Oversight and monitoring

### Composition of the data monitoring committee, its role and reporting structure {21a}

Due to the short duration of the trial and known minimal risks, a data monitoring committee is not needed.

### Adverse event reporting and harms {22}

Hydroxychloroquine use may have potential risks for the participants. It is possible that the ingestion of the drug causes side effects (loss of appetite, emotional instability, headache, blurred vision, pain in the belly, nausea, itching, and spots on the skin), described in the informed consent. To monitor these side effects, follow-up phone calls will be made every four weeks and adverse events will be investigated for open-ended questions, including general symptoms, such as headache, nausea, and blurred vision. An adverse event that meets the criteria for a serious adverse will be reported to the institutional ethics committee. A serious adverse event for this study is any untoward medical occurrence that is believed by the investigators to be causally related to study-drug and results in any of the following: life-threatening condition, severe/permanent disability, or prolonged hospitalization

### Frequency and plans for auditing trial conduct {23}

There is no plan for auditing trial conduct.

### Plans for communicating important protocol amendments to relevant parties (e.g., trial participants, ethical committees) {25}

Important protocol changes as well as changes in eligibility criteria, outcomes, or analyses will be communicated to the investigators, institutional ethics committee, trial participants, and trial registries.

### Dissemination plans {31a}

The investigators will communicate relevant trial results to participants by telephone call and printed material. The publication in a peer-reviewed journal is mandatory for the thesis author to have her PhD title acknowledged. The results database will be made available at the university repository.

## Discussion

The COVID pandemics forced changes in protocol. The research center was closed and volunteers were instructed to cancel the visits. This incurred in the loss of 4 subjects. An interim analysis was performed to check for the need to continue recruiting. A new sample size calculation was performed. The large number necessary to obtain 80% power made the project unviable. The investigators decided to abandon the project as initially designed. The finding of a small but significant reduction in the OSA severity may be hypothesis-generating. We may continue searching for possible inflammatory mechanisms and therapeutic targets of OSA.

## Trial status

The recruitment began on 03/19. Due to the changes in protocol during the COVID pandemic, recruitment will not be resumed.

## Data Availability

Not applicable.

## Abbreviations

HCQ: Hydroxychloroquine
OSA: Obstructive sleep apnea
NO: Nitric oxide
FMD: Flow-mediated dilation
PAT: Peripheral artery tonometry
RHI: Reactive hyperemia index
TNF-α: Tumor necrosis factor alpha

## References

[1] Senaratna CV, Perret JL, Lodge CJ, Lowe AJ, Campbell BE, Matheson MC, Hamilton GS, Dharmage SC (2017). Prevalence of obstructive sleep apnea in the general population: a systematic review. Sleep Med Rev..

[2] Tufik S, Santos-Silva R, Taddei JA, Bittencourt LR (2010). Obstructive sleep apnea syndrome in the Sao Paulo Epidemiologic Sleep Study. Sleep Med..

[3] Dong JY, Zhang YH, Qin LQ (2013). Obstructive sleep apnea and cardiovascular risk: meta-analysis of prospective cohort studies. Atherosclerosis..

[4] Mansur, Antonio de Padua, & Favarato, Desidério. Mortalidade por doenças cardiovasculares no Brasil e na região metropolitana de São Paulo: atualização 2011. Arquivos Brasileiros de Cardiologia. 2012; 10.1590/S0066-782X2012005000061

[5] Wang X, Ouyang Y, Wang Z, Zhao G, Liu L, Bi Y (2013). Obstructive sleep apnea and risk of cardiovascular disease and all-cause mortality: a meta-analysis of prospective cohort studies. Int J Cardiol..

[6] Peppard PE, Young T, Palta M, Skatrud J (2000). Prospective study of the association between sleep-disordered breathing and hypertension. N Engl J Med..

[7] Hayashi M, Fujimoto K, Urushibata K, Uchikawa S, Imamura H, Kubo K (2003). Nocturnal oxygen desaturation correlates with the severity of coronary atherosclerosis in coronary artery disease. Chest..

[8] Yaggi HK, Concato J, Kernan WN, Lichtman JH, Brass LM, Mohsenin V (2005). Obstructive sleep apnea as a risk factor for stroke and death. N Engl J Med..

[9] Quan SF, Gersh BJ, National Center on Sleep Disorders Research; National Heart, Lung, and Blood Institute. Cardiovascular consequences of sleep-disordered breathing: past, present and future: report of a workshop from the National Center on Sleep Disorders Research and the National Heart, Lung, and Blood Institute. Circulation. 2004. 10.1161/01.CIR.0000118216.84358.22. 10.1161/01.CIR.0000118216.84358.22 14993147

[10] Somers VK, White DP, Amin R, Abraham WT, Costa F, Culebras A, et al. Sleep apnea and cardiovascular disease: an American Heart Association/american College Of Cardiology Foundation Scientific Statement from the American Heart Association Council for High Blood Pressure Research Professional Education Committee, Council on Clinical Cardiology, Stroke Council, and Council On Cardiovascular Nursing. In collaboration with the National Heart, Lung, and Blood Institute National Center on Sleep Disorders Research (National Institutes of Health). Circulation. 2008. 10.1161/CIRCULATIONAHA.107.189375.

[11] Gilat H, Vinker S, Buda I, Soudry E, Shani M, Bachar G (2014). Obstructive sleep apnea and cardiovascular comorbidities: a large epidemiologic study. Medicine (Baltimore)..

[12] Lamberts M, Nielsen OW, Lip GY, Ruwald MH, Christiansen CB, Kristensen SL, et al. Cardiovascular risk in patients with sleep apnoea with or without continuous positive airway pressure therapy: follow-up of 4.5 million Danish adults. J Intern Med. 2014. 10.1111/joim.12302. 10.1111/joim.12302 25169419

[13] Martinez D, Klein C, Rahmeier L, da Silva RP, Fiori CZ, Cassol CM, Gonçalves SC, Bos AJ (2012). Sleep apnea is a stronger predictor for coronary heart disease than traditional risk factors. Sleep Breath..

[14] Augustin HG, Kozian DH, Johnson RC. Differentiation of endothelial cells: analysis of the constitutive and activated endothelial cell phenotypes. Bioessays. 1994. 10.1002/bies.950161208. 10.1002/bies.950161208 7840769

[15] Camici GG, Savarese G, Akhmedov A, Lüscher TF (2015). Molecular mechanism of endothelial and vascular aging: implications for cardiovascular disease. Eur Heart J..

[16] Park KH, Park WJ (2015). Endothelial Dysfunction: Clinical Implications in Cardiovascular Disease and Therapeutic Approaches. J Korean Med Sci..

[17] Thijssen DH, Black MA, Pyke KE, Padilla J, Atkinson G, Harris RA, Parker B, Widlansky ME, Tschakovsky ME, Green DJ (2011). Assessment of flow-mediated dilation in humans: a methodological and physiological guideline. Am J Physiol Heart Circ Physiol..

[18] Xu Y, Arora RC, Hiebert BM, Lerner B, Szwajcer A, McDonald K, Rigatto C, Komenda P, Sood MM, Tangri N (2014). Non-invasive endothelial function testing and the risk of adverse outcomes: a systematic review and meta-analysis. Eur Heart J Cardiovasc Imaging..

[19] Greyling A, van Mil AC, Zock PL, Green DJ, Ghiadoni L, Thijssen DH, et al. Adherence to guidelines strongly improves reproducibility of brachial artery flow-mediated dilation. Atherosclerosis. 2016. 10.1016/j.atherosclerosis.2016.03.011. 10.1016/j.atherosclerosis.2016.03.011 27023841

[20] Bonetti PO, Pumper GM, Higano ST, Holmes DR, Kuvin JT, Lerman A (2004). Noninvasive identification of patients with early coronary atherosclerosis by assessment of digital reactive hyperemia. J Am Coll Cardiol..

[21] Bruno RM, Gori T, Ghiadoni L. Endothelial function testing and cardiovascular disease: focus on peripheral arterial tonometry. Vasc Health Risk Manag. 2014. 10.2147/VHRM.S44471. 10.2147/VHRM.S44471 PMC4196841 25328403

[22] Matsuzawa Y, Sugiyama S, Sumida H, Sugamura K, Nozaki T, Ohba K, Matsubara J, Kurokawa H, Fujisue K, Konishi M, Akiyama E, Suzuki H, Nagayoshi Y, Yamamuro M, Sakamoto K, Iwashita S, Jinnouchi H, Taguri M, Morita S, Matsui K, Kimura K, Umemura S, Ogawa H (2013). Peripheral endothelial function and cardiovascular events in high-risk patients. J Am Heart Assoc..

[23] Matsue Y, Suzuki M, Nagahori W, Ohno M, Matsumura A, Hashimoto Y, Yoshida K, Yoshida M (2013). Endothelial dysfunction measured by peripheral arterial tonometry predicts prognosis in patients with heart failure with preserved ejection fraction. Int J Cardiol..

[24] Rubinshtein R, Kuvin JT, Soffler M, Lennon RJ, Lavi S, Nelson RE, Pumper GM, Lerman LO, Lerman A (2010). Assessment of endothelial function by non-invasive peripheral arterial tonometry predicts late cardiovascular adverse events. Eur Heart J..

[25] Brant LC, Barreto SM, Passos VM, Ribeiro AL (2013). Reproducibility of peripheral arterial tonometry for the assessment of endothelial function in adults. J Hypertens..

[26] Hamburg NM, Palmisano J, Larson MG, Sullivan LM, Lehman BT, Vasan RS, Levy D, Mitchell GF, Vita JA, Benjamin EJ (2011). Relation of brachial and digital measures of vascular function in the community: the Framingham heart study. Hypertension..

[27] Schnabel RB, Schulz A, Wild PS, Sinning CR, Wilde S, Eleftheriadis M, et al. Noninvasive vascular function measurement in the community: cross-sectional relations and comparison of methods. Circ Cardiovasc Imaging. 2011. 10.1161/CIRCIMAGING.110.961557. 10.1161/CIRCIMAGING.110.961557 21551420

[28] Yilmaz Avci A, Avci S, Lakadamyali H, Can U (2017). Hypoxia and inflammation indicate significant differences in the severity of obstructive sleep apnea within similar apnea-hypopnea index groups. Sleep Breath..

[29] Unnikrishnan D, Jun J, Polotsky V (2015). Inflammation in sleep apnea: an update. Rev Endocr Metab Disord..

[30] Wang J, Yu W, Gao M, Zhang F, Gu C, Yu Y, et al. Impact of Obstructive Sleep Apnea Syndrome on Endothelial Function, Arterial Stiffening, and Serum Inflammatory Markers: An Updated Meta-analysis and Metaregression of 18 Studies. J Am Heart Assoc. 2015;4(11). 10.1161/JAHA.115.002454. 10.1161/JAHA.115.002454 PMC4845236 26567373

[31] Hoyos CM, Melehan KL, Liu PY, Grunstein RR, Phillips CL (2015). Does obstructive sleep apnea cause endothelial dysfunction? A critical review of the literature. Sleep Med Rev..

[32] Hoffmann M, Wolf J, Szyndler A, Singh P, Somers VK, Narkiewicz K (2017). Serum of obstructive sleep apnea patients impairs human coronary endothelial cell migration. Arch Med Sci..

[33] da Rosa DP, Forgiarini LF, Silva MB e, Fiori CZ, Andrade CF, Martinez D, et al. Antioxidants inhibit the inflammatory and apoptotic processes in an intermittent hypoxia model of sleep apnea. Inflamm Res. 2015. 10.1007/s00011-014-0778-5. 10.1007/s00011-014-0778-5 25380745

[34] Klein C, Martinez D, Hackenhaar FS, Medeiros TM, Marcolin ML, Silveira FS, Wainstein MV, Gonçalvez SC, Benfato MS (2010). Carbonyl groups: Bridging the gap between sleep disordered breathing and coronary artery disease. Free Radic Res..

[35] Kuznik A, Bencina M, Svajger U, Jeras M, Rozman B, Jerala R (2011). Mechanism of endosomal TLR inhibition by antimalarial drugs and imidazoquinolines. J Immunol..

[36] Edfeldt K, Swedenborg J, Hansson GK, Yan ZQ (2002). Expression of toll-like receptors in human atherosclerotic lesions: a possible pathway for plaque activation. Circulation..

[37] Jang CH, Choi JH, Byun MS, Jue DM (2006). Chloroquine inhibits production of TNF-alpha, IL-1beta and IL-6 from lipopolysaccharide-stimulated human monocytes/macrophages by different modes. Rheumatology (Oxford)..

[38] Weber SM, Levitz SM (2000). Chloroquine interferes with lipopolysaccharide-induced TNF-alpha gene expression by a nonlysosomotropic mechanism. J Immunol..

[39] Jacobsson LT, Turesson C, Gülfe A, Kapetanovic MC, Petersson IF, Saxne T, Geborek P (2005). Treatment with tumor necrosis factor blockers is associated with a lower incidence of first cardiovascular events in patients with rheumatoid arthritis. J Rheumatol..

[40] Tang C, Godfrey T, Stawell R, Nikpour M (2012). Hydroxychloroquine in lupus: emerging evidence supporting multiple beneficial effects. Intern Med J..

[41] Lesiak A, Narbutt J, Sysa-Jedrzejowska A, Lukamowicz J, McCauliffe DP, Wózniacka A (2010). Effect of chloroquine phosphate treatment on serum MMP-9 and TIMP-1 levels in patients with systemic lupus erythematosus. Lupus..

[42] Newby AC (2015). Metalloproteinases promote plaque rupture and myocardial infarction: A persuasive concept waiting for clinical translation. Matrix Biol..

[43] Wallace DJ, Metzger AL, Stecher VJ, Turnbull BA, Kern PA (1990). Cholesterol-lowering effect of hydroxychloroquine in patients with rheumatic disease: reversal of deleterious effects of steroids on lipids. Am J Med..

[44] Morris SJ, Wasko MC, Antohe JL, Sartorius JA, Kirchner HL, Dancea S, Bili A (2011). Hydroxychloroquine use associated with improvement in lipid profiles in rheumatoid arthritis patients. Arthritis Care Res (Hoboken)..

[45] Kerr G, Aujero M, Richards J, Sayles H, Davis L, Cannon G, Caplan L, Michaud K, Mikuls T (2014). Associations of hydroxychloroquine use with lipid profiles in rheumatoid arthritis: pharmacologic implications. Arthritis Care Res (Hoboken)..

[46] Petri M, Lakatta C, Magder L, Goldman D (1994). Effect of prednisone and hydroxychloroquine on coronary artery disease risk factors in systemic lupus erythematosus: a longitudinal data analysis. Am J Med..

[47] Solomon DH, Massarotti E, Garg R, Liu J, Canning C, Schneeweiss S (2011). Association between disease-modifying antirheumatic drugs and diabetes risk in patients with rheumatoid arthritis and psoriasis. JAMA..

[48] Bili A, Sartorius JA, Kirchner HL, Morris SJ, Ledwich LJ, Antohe JL, Dancea S, Newman ED, Wasko MC (2011). Hydroxychloroquine use and decreased risk of diabetes in rheumatoid arthritis patients. J Clin Rheumatol..

[49] Wasko MC, Hubert HB, Lingala VB, Elliott JR, Luggen ME, Fries JF, Ward MM (2007). Hydroxychloroquine and risk of diabetes in patients with rheumatoid arthritis. JAMA..

[50] Rekedal LR, Massarotti E, Garg R, Bhatia R, Gleeson T, Lu B, Solomon DH (2010). Changes in glycosylated hemoglobin after initiation of hydroxychloroquine or methotrexate treatment in diabetes patients with rheumatic diseases. Arthritis Rheum..

[51] Wasko MC, McClure CK, Kelsey SF, Huber K, Orchard T, Toledo FG (2015). Antidiabetogenic effects of hydroxychloroquine on insulin sensitivity and beta cell function: a randomised trial. Diabetologia..

[52] Quatraro A, Consoli G, Magno M, Caretta F, Nardozza A, Ceriello A, et al. Hydroxychloroquine in decompensated, treatment-refractory noninsulin-dependent diabetes mellitus. A new job for an old drug? Ann Intern Med. 1990. 10.7326/0003-4819-112-9-678. 10.7326/0003-4819-112-9-678 2110430

[53] Gerstein HC, Thorpe KE, Taylor DW, Haynes RB (2002). The effectiveness of hydroxychloroquine in patients with type 2 diabetes mellitus who are refractory to sulfonylureas--a randomized trial. Diabetes Res Clin Pract..

[54] Petri M (2011). Use of hydroxychloroquine to prevent thrombosis in systemic lupus erythematosus and in antiphospholipid antibody-positive patients. Curr Rheumatol Rep..

[55] Achuthan S, Ahluwalia J, Shafiq N, Bhalla A, Pareek A, Chandurkar N, Malhotra S (2015). Hydroxychloroquine's Efficacy as an Antiplatelet Agent Study in Healthy Volunteers: A Proof of Concept Study. J Cardiovasc Pharmacol Ther..

[56] Edwards MH, Pierangeli S, Liu X, Barker JH, Anderson G, Harris EN (1997). Hydroxychloroquine reverses thrombogenic properties of antiphospholipid antibodies in mice. Circulation..

[57] Shukla AM, Bose C, Karaduta OK, Apostolov EO, Kaushal GP, Fahmi T, Segal MS, Shah SV (2015). Impact of Hydroxychloroquine on Atherosclerosis and Vascular Stiffness in the Presence of Chronic Kidney Disease. PLoS One..

[58] Jung H, Bobba R, Su J, Shariati-Sarabi Z, Gladman DD, Urowitz M, Lou W, Fortin PR (2010). The protective effect of antimalarial drugs on thrombovascular events in systemic lupus erythematosus. Arthritis Rheum..

[59] Sharma TS, Wasko MC, Tang X, Vedamurthy D, Yan X, Cote J, et al. Hydroxychloroquine Use Is Associated With Decreased Incident Cardiovascular Events in Rheumatoid Arthritis Patients. J Am Heart Assoc. 2016;5(1). 10.1161/JAHA.115.002867. 10.1161/JAHA.115.002867 PMC4859400 26727968

