# Correction to: Effects of Hydroxychloroquine on endOthelial function in eLDerly with sleep apnea (HOLD): study protocol for a randomized clinical trial

**DOI:** 10.1186/s13063-021-05683-x

**Published:** 2021-10-18

**Authors:** Leticia Maria Tedesco Silva, Antonio Cortes, Beatriz Rossi, Liliana Boll, Gustavo Waclawovsky, Bruna Eibel, Sandro Cadaval Gonçalves, Maria Claudia Irigoyen, Denis Martinez

**Affiliations:** 1 Universidade Federal do Rio Grande do Sul, Porto Alegre, Brazil; 2 Instituto de Cardiologia - Fundação Universitária de Cardiologia (IC-FUC), Porto Alegre, Brazil; 3 Universidade de São Paulo, São Paulo, Brazil

**Correction to: Trials 22, 638 (2021)**

**https://doi.org/10.1186/s13063-021-05610-0**

Following the publication of the original article [1], we were notified of a spelling error in the name of author Gustavo Waclawovsky (missing letter “v”).

- Originally published name: Gustavo Waclawosky
- Corrected name: Gustavo Waclawovsky

The original article has been corrected.
